# S100A2 is strongly expressed in airway basal cells, preneoplastic bronchial lesions and primary non-small cell lung carcinomas

**DOI:** 10.1038/sj.bjc.6602188

**Published:** 2004-10-05

**Authors:** S L Smith, M Gugger, P Hoban, D Ratschiller, S G Watson, J K Field, D C Betticher, J Heighway

**Affiliations:** 1 Gene Function Group, Roy Castle Lung Cancer Programme (Clinical Dental Sciences), University of Liverpool Cancer Research Centre, 200 London Road, Liverpool L3 9TA, UK; 2 Institute of Pathology, University of Bern, 3010 Bern, Switzerland; 3 Institute of Science and Technology in Medicine, Keele University School of Medicine, University Hospital of North Staffordshire, Stoke-on-Trent ST4 7QB, UK; 4 Institute of Medical Oncology, University of Bern, 3010 Bern, Switzerland

**Keywords:** lung, carcinoma, S100A2, preneoplasia, expression

## Abstract

S100A2 gene products were shown to be frequently and dramatically over-represented in non-small cell lung cancer (NSCLC) lesions over normal tissue by microarray analysis. We have now analysed an independent series of NSCLC tumours and multiple matched normal bronchial epithelial sites by RT–PCR and immunohistochemistry to investigate: whether this expression pattern can be confirmed and whether elevated expression is associated with tumour histology, clinical outcome or preneoplasia. In this second series, S100A2 was strongly expressed in 76% (35 out of 46) of tumours, more frequently in squamous cell than adenocarcinomas (*P*<0.002). This strong expression was not related to high-level gene amplification, but was associated in one of five informative cases with an allele-specific imbalance in transcript levels. Most tumours strongly expressed the ΔNp63 transcript, the product of which is a putative regulator of S100A2 transcription and while all but one of the tumours positive for ΔNp63 expressed S100A2, others negative for this regulator also expressed the gene. Contrary to the hypothesis that S100A2 is a tumour suppressor, no somatic mutations were identified in the coding sequence in 44 tumours. Furthermore, an examination of multiple tumour-free epithelial sites from 20 patients showed that strong expression was often associated with increasing levels of disorder in preinvasive bronchial lesions (*P*<0.0001).

Lung cancer is the leading cause of cancer-related death. The 5-year survival rate in Europe is poor, remaining at approximately 10% (Bray *et al*, 2002). This is a consequence of a generally late presentation coupled with an inability to treat disseminated disease effectively. It has been hypothesised that the early presymptomatic detection of lung cancer and early intervention would significantly reduce associated mortality. However, we must bear in mind that it is unlikely that the use of conventional, toxic chemotherapeutic agents or radical surgery will be justified unless a certainty of future life-threatening disease can be established. Successful early detection methods will therefore need to be coupled to minimally toxic treatment (chemoprevention) strategies. The examination of the expression of particular genes in tumour compared to normal cells is one way in which new disease biomarkers and novel therapeutic targets can be identified.

Dual-channel cDNA microarray analysis of relative expression in 39 primary non-small cell lung carcinomas (NSCLC) compared to matched normal tissue was used to construct ranked lists of the most frequently and dramatically differentially expressed genes (Heighway *et al*, 2002). One of the sequences so highlighted, S100A2, a gene considered to be a tumour suppressor, was found to be over-represented in tumour samples compared to matched normal lung. S100A2 is one of a family of calcium binding proteins characterised by two conserved calcium binding EF-hand domains (Donato, 1999). The S100A2 gene is located within the epidermal differentiation complex (EDC) on chromosome 1q21 and is in a region that is frequently rearranged in many types of human cancer (Ilg *et al*, 1996), including NSCLC (Whang-Peng *et al*, 1991). Originally discovered through subtractive hybridisation, S100A2 was reportedly downregulated in malignant breast epithelial cells compared to normal mammary epithelial cells (Lee *et al*, 1991). Apparent under-representation or downregulation of the gene product in breast cancer cells relative to normal breast epithelia has subsequently been confirmed by a number of groups (Pedrocchi *et al*, 1994; Wicki *et al*, 1997; Liu *et al*, 2000). Diminished S100A2 expression has also been described in a cell line model study of early lung cancer (Feng *et al*, 2001) and a number of other cancers, including head and neck cancer (Nagy *et al*, 2001) and skin melanoma (Maelandsmo *et al*, 1997). Conversely, in addition to our analysis of NSCLC, over-representation of S100A2 has also been reported in gastric cancer (El-Rifai *et al*, 2002) ovarian cancer (Hough *et al*, 2001) and head and neck cancer (Villaret *et al*, 2000).

At a superficial level, these results appear to suggest contradictory patterns of gene expression between different tissues and diseases and in some cases, between different centres. There is little doubt (based on our earlier transcript and protein-based analyses: Heighway *et al*, 2002) that S100A2 is very strongly expressed in the majority of primary NSCLC lesions. In an attempt to explore whether high-level expression is either normal or associated with carcinogenesis, we have carried out a series of immunohistochemical analyses on an independent series of tumour and multiple matched normal and preneoplastic bronchial epithelial sites from a panel of NSCLC patients. We have supplemented this analysis with: a re-examination of transcript levels in this new series of lesions, a mutation scan of the gene in primary tumours and tumour cell lines and an investigation of allele-specific expression in normal and tumour tissue. Finally, by comparative multiplex RT–PCR (cmRT–PCR), we have examined the expression of ΔNp63 in the tissue samples following the recent suggestion that S100A2 is a target of the ΔNp63 oncogenic pathway (Hibi *et al*, 2003) and bearing in mind that the p63 transcript was also highlighted in our microarray-generated, tumour over-represented gene lists (>two-fold in 24%, nine out of 37 patients).

## MATERIALS AND METHODS

### Patients and cell lines

Tissues from a total of 48 patients with resectable-staged NSCLC were used in the expression and immunohistochemical (IHC) analyses (37 men, 11 women, median age 65.3 years, range 30.6–80.7). Frozen tumour tissue was obtained from all patients and matched normal lung from all but two patients. A total of 17 patients had stage I disease, 12 stage II and 19 stage III or above. All tumours were classified according to the standard criteria of the WHO. There were 20 squamous cell carcinomas, 24 adenocarcinomas, three large cell carcinomas and one poorly differentiated NSCLC. Matched normal tissues were obtained from bronchial epithelium at a tumour-free site. Frozen tissues were kept at −70°C. Blocks for immunohistochemistry were fixed in formalin and embedded in paraffin. For 20 of the patients in this series, blocks of matched histologically normal bronchial epithelia from a further five independent, tumour-free sites were also collected. Tumour DNA samples from a further 38 UK NSCLC patients, including material derived from samples previously screened by microarray was scanned for somatic mutations. Lung carcinoma cell lines: CORL23, CRL5082, DMS53, SKLU1, HTB182, A549, LUDL1, CORL88 were obtained from ATCC. The analysis of lung cancer-related gene expression in the clinical samples was approved by local ethics committees in Bern and Liverpool.

### RNA and DNA extraction

Total RNA was extracted from 20 40-*μ*m sections of frozen tumour and matched normal tissue using a standard TRIzol (Life Technologies) protocol (Heighway *et al*, 2003). DNA was extracted from 10 40-*μ*m frozen sections, using a standard SDS lysis/phenol extraction procedure (Heighway *et al*, 2003). Sections of 6 *μ*m were cut adjacent to both RNA and DNA sections and stained with haematoxylin and eosin to show tumour histology and to estimate the overall percentage of overt viable tumour cells.

### PCR and RT–PCR

PCR reactions of 50 *μ*l containing approximately 50 ng of DNA, 250 ng of each primer, 1 × *Taq* reaction buffer (Roche), 200 *μ*M dNTPs (Roche) and 1 U of *Taq* polymerase were assembled and cycled 30 times, in a PE Applied Biosystems GeneAmp PCR System 9700 machine, at 58°C for 1 min, 74°C for 1 min and 94°C for 1 min, with an initial denaturation of 2 min at 94°C and two final incubations of 58°C for 1 min and 74°C for 3 min. Reverse transcription was performed with the Reverse Transcription System from Promega, in a 20 *μ*l poly-T-primed synthesis using approximately 500 ng of total RNA. RT–PCR reactions were assembled and cycled as for PCR, except that a 1 *μ*l aliquot of cDNA was used instead of DNA.

### Primers

Transcript-specific primers SA21a (5′ GAA CTT CTG CAC AAG GAG CTG) and SA21b (5′ AAA GGC ATC AAC AGT CCT GGG) were used to amplify S100A2 from cDNA and p63F1 (5′ AGT GTG CTG GTA CCT TAT GAG) and p63R3 (5′ GTA CTG TCC GAA ACT TGC TGC T) were used to amplify p63 transcripts. Transcript-specific primers for the nondifferentially-expressed control gene amyloid beta precursor protein binding protein (KIAA0228, Accession number NM_006380) APPBP2F (5′ GAA CTG TGT GCA CTC CTA TTT G) and APPBP2R (5′ CCG TGC CAA ATA CAC TGC ATG T) were used in cmRT–PCR.

Primers pSA21a (5′ CTG GCT TGG TGA TGA GTG TAT) and pSA21b (5′ CTC AAA GGC ATC AAC AGT CCT), were used to amplify the S100A2 gene from DNA for RFLP analysis. Exons 2 and 3 were amplified from tumour genomic DNA for WAVE analysis using SA2F (5′ CAG AAG TGG ATC CTA CAG GCT) and SA2R (5′ ACT GGG GGA GAG ATT TAA CCT G), and SA3F (5′ GCT GGC TTG GTG ATG AGT GTA) and SA3R (5′ GGT TAT GGA ACA TCA CTG AGC A), respectively.

### Comparative multiplex RT–PCR

Comparative multiplex (cm) RT–PCR was used to compare levels of test gene transcripts between normal lung and tumour samples, as described previously (Heighway *et al*, 2002; Smith *et al*, 2003). Briefly, the test gene transcript was coamplified with a nondifferentially expressed control transcript (APPBP2 – KIAA0228) in the same reaction. Reaction products were run on 3% agarose gels containing 0.5 *μ*g ml^−1^ ethidium bromide, in 0.5 × TBE and visualised by UV illumination. Relative expression was quantified on an Agilent 2100 Bioanalyser using DNA 1000 chips (Agilent Biotechnologies). To obtain values for S100A2 and ΔNp63 expression relative to the control transcript APPBP2, peak heights of the test genes were divided by those of the control gene within each sample. The comparative value in the patient tumour tissue was then divided by that from the matched normal to calculate the fold difference in expression of the test gene in each patient. Where no test-gene product was seen in normal tissue and where a product was detected in tumour, the fold-change was therefore infinite. While in many cases test gene expression was not detected in normal lung, a differential expression of the test gene of ⩾two-fold in the tumour was considered to indicate a relative over-representation of that gene in the tumour. It should be noted that this procedure is only semiquantitative and does not reflect an absolute expression level of the test gene mRNA.

### RFLP-RT–PCR and RFLP-PCR

A 10 *μ*l aliquot of PCR or RT–PCR product was digested with 5 U of *Hinf*I (NEB) in a 40 *μ*l reaction containing 1 × manufacturer's restriction enzyme buffer at 37°C for 3 h. Digests were visualised by gel electrophoresis and quantified by running on DNA 100 LabChips® in an Agilent 2100 Bioanalyser (Agilent Technologies).

### WAVE mutation analysis

PCR products from S100A2 exons 2 and 3 were screened for mutation using denaturing high-performance liquid chromatography (O'Donovan *et al*, 1998) using a WAVE® system (Model 3500A Transgenomics Limited, Crewe, UK). To account for possible deletions of 1q21 in tumours preventing heteroduplex formation, PCR products were mixed 3 : 1 ratio with PCR product from normal wild-type DNA. PCR products were denatured at 94°C and returned to 25°C (at 1°C min^−1^) for heteroduplex formation. Denaturing acetonitrile gradient profiles and melting temperatures for analysis were determined using Wavemaker™ 4.1 software (Transgenomics). Exon 2 was analysed at 62.5°C on a gradient of 55% buffer B (Transgenomics; 25% acetonitrile/0.1 M TEAA) for 4 min. Exon 3 was analysed at 60°C on a gradient of 56% buffer B for 4 min.

### Immunohistochemical analysis

Formalin-fixed, paraffin-embedded sections of 5 *μ*m were dewaxed in xylene for 30 min, followed by rehydration through a series of graded ethanols to water. Antigens were retrieved using a 15 min microwave treatment in Vector unmasking solution (10 mM sodium citrate buffer, pH 6). Slides were left to stand in the unmasking solution for 15 min after treatment and then placed under running water for 5 min prior to loading onto a Dako Autostainer. Endogenous peroxidase was blocked by a 15 min incubation in 3% v v^−1^ H_2_O_2_. Sections were then incubated for 60 min in a 1 in 500 dilution of S100A2 rabbit polyclonal antibody (Dako). This was followed by detection of specific staining with the Dako LSAB2 HRP system with DAB (diaminobenzidine) substrate. Slides were counterstained in Haematoxylin/Scotts tap water and dehydrated through a series of graded ethanols to xylene. Sections were mounted in DPX and visualised. In negative control sections, the primary antibody was replaced with diluent. A tumour which strongly expressed S100A2 was used as a positive control on all autostainer runs.

The sections were reviewed by our pathologist (MG) in a blinded fashion, considering histology, grading of differentiation and S100A2 staining. Epithelial changes were graded according to the criteria reviewed by Travis *et al*, 1999. Squamous metaplasia is the first clear-cut morphological change in the respiratory epithelium induced by smoking. We grouped epithelial sites into non-neoplastic (histologically normal and hyperplastic) and premalignant/noninvasive (metaplastic, dysplastic and carcinoma *in situ*). In detail, regarding S100A2 staining the following criteria were used for cytoplasmic and/or nuclear staining, for the intensity −, +, ++, +++ and for the frequency 0–5, 5–50, 50–100%. For data grouping, a tumour (or bronchial site) was considered to be positive for nuclear and cytoplasmic staining if >5% of the (site: apical epithelial) cells were scored with a ++ to +++ intensity.

### Statistical analysis

χ^2^-analysis was used to compare the frequency of nuclear and cytoplasmic staining for S100A2 in: normal (normal and hyperplastic) *vs* preneoplastic/noninvasive (metaplastic/dysplastic/CIS) epithelia and between squamous cell and adenocarcinoma. The comparison of tumour expression between RT–PCR and IHC was carried out using a Mann–Whitney test with fold overexpression as a continuous variable and staining considered as positive or negative. Patients were further placed into two groups according to their S100A2 expression (IHC) and overall survival and remission duration were analysed by Kaplan–Meier survivor function estimates.

## RESULTS

### S100A2 expression in NSCLC

**Figure 1:**
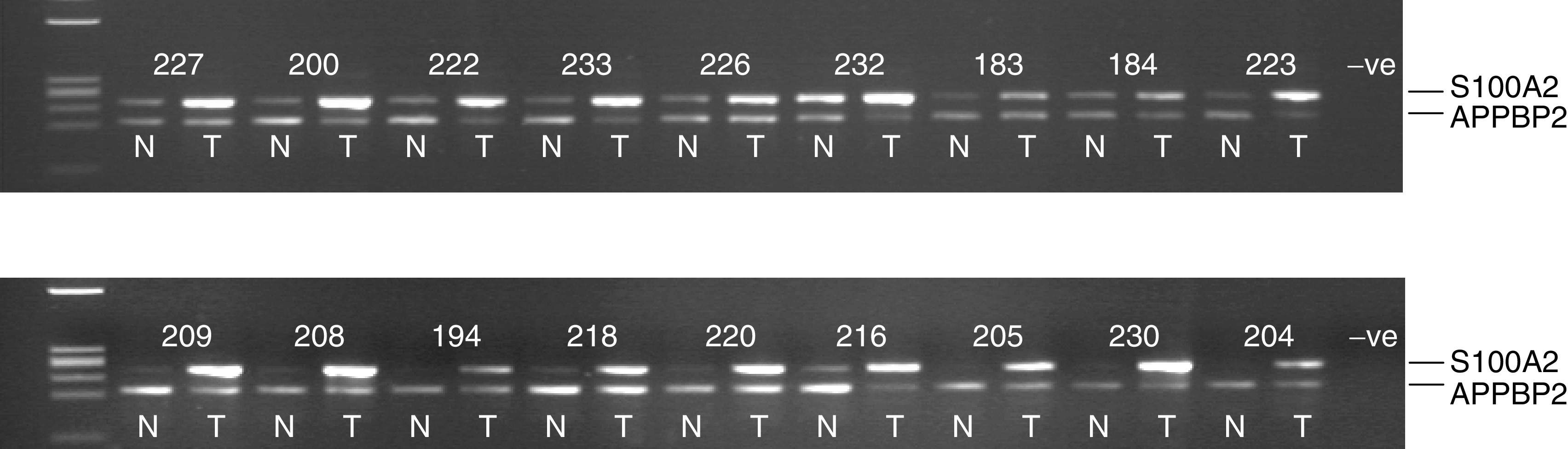
Expression of S100A2 in primary NSCLC. S100A2 transcripts were coamplified with transcripts of a control gene by comparative multiplex RT–PCR. Representative agarose gels are shown. Transcripts of the gene for APPBP2 were used as the control. N=normal bronchial epithelial tissue. T=NSCLC tissue. Numbers represent anonymised patient numbers. Marker track (extreme left on all agarose gel images) is a ΦX174/*Hae*III digest.

Building on our earlier microarray validation study, we have further investigated the expression of S100A2 in an independent series of NSCLC patients. Levels of S100A2 transcript in tumour samples of this new series were compared to those in matched normal lung, by cmRT–PCR. An endogenous control transcript (ABPPBP2) previously identified as not differentially expressed between normal lung and lung tumours (Heighway *et al*, 2002) was used as an internal control transcript. Results of this analysis are illustrated in Figure 1

.

S100A2 expression was detected in the normal tissue of most, but not all patients. However, most tumours showed an over-representation of the transcript relative to the matched normal tissue levels. When cmRT–PCR products were run on an Agilent Bioanalyser, 35 out of 46 (76%) patients showed a ⩾two-fold over-representation of S100A2 in their tumour sample compared to their matched normal lung sample (Table 1

**Table 1:** Semiquantitative analysis of S100A2 and ΔNp63 transcript expression in NSCLC patients

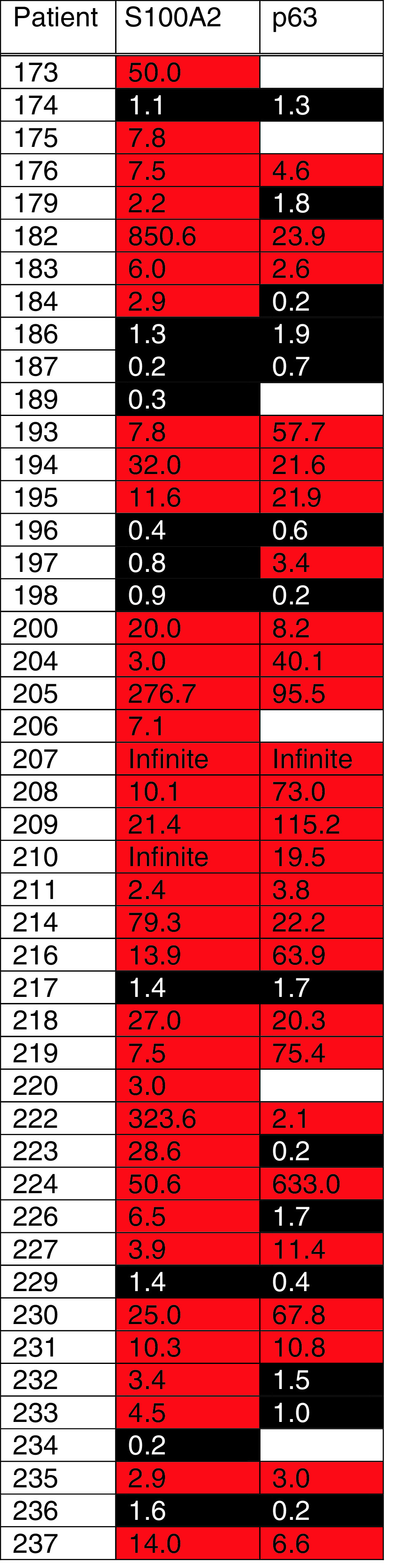

S100A2 or ΔNp63 transcripts were coamplified with a control gene transcript from normal and tumour tissue of NSCLC patients, by cmRT–PCR. Reaction products were run on an Agilent bioanalyser. Peak heights of control and test genes were compared for each patient to generate a semiquantitative value for the fold expression of the test gene transcript in the tumour sample compared to matched normal lung. Red boxes indicate where a patient showed ⩾two-fold expression of the test transcript in the tumour sample compared to normal lung. Black boxes indicate a <two-fold expression and a white box indicates no data for that patient.

).

In certain cases, we detected an additional transcript in the patients' tumour sample. By cloning and sequence analysis, we determined that an additional exon (exemplified by BM016868) was spliced into this transcript, between exons 2 and 3 (called exon 2A). Using exon-specific RT–PCR, this alternative transcript could be specifically amplified from a range of tumours but not normal lung. However, the levels as judged by cmRT–PCR were invariably low in comparison to the normal exon 2/3 gene product (results not shown). We therefore excluded further consideration of this alternatively spliced transcript from this study.

### Mutation analysis

**Figure 2:**
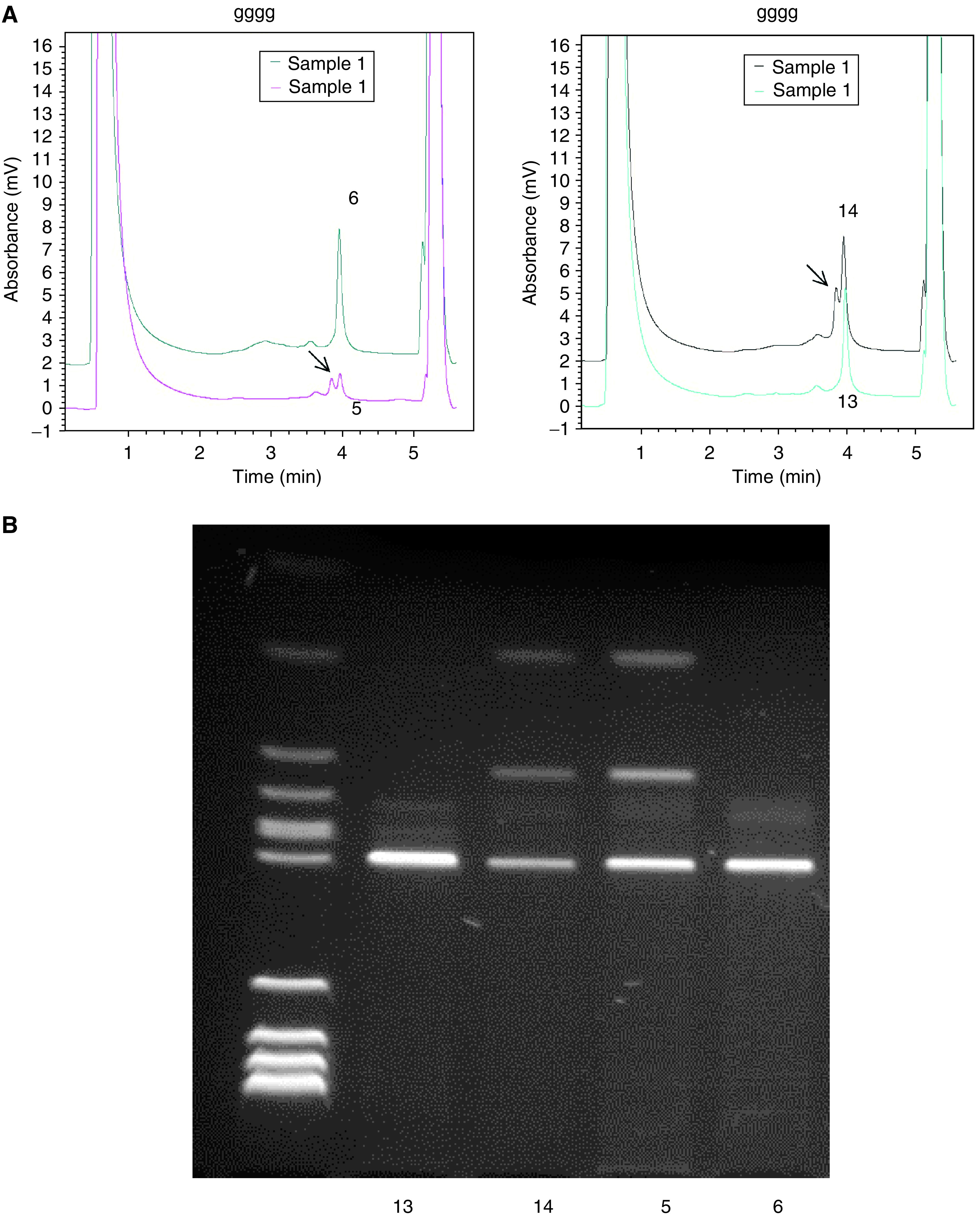
Identification of S100A2 exon 3 polymorphism. (**A**) WAVE analysis of exon 3 in lung tumours: 5 and 6 left panel, 13 and 14 right panel. Arrows identify heteroduplex fragments. (**B**) RFLP analysis of exon 3 in lung tumours 13, 14, 5 and 6. Digestion with *Hinf*I reveals the presence of the polymorphism in tumours 5 and 14.

It has been suggested that S100A2 is a tumour suppressor gene and that its expression is downregulated in neoplastic cells. If that were the case, it is possible that the gene would be the target of somatic mutational inactivation events. Similarly, while we observed strong expression in the majority of NSCLC samples, it is possible that this apparently elevated transcript level was driven by upregulation of a functionally inactive protein in the tumour cell. Conversely, if the gene were inappropriately activated in a tumour cell thereby providing an oncogenic stimulus, at least in some instances this might conceivably be achieved by point mutation of the coding sequence. To explore these different possibilities, we carried out a mutational screen of the S100A2 coding sequence (which is contained within exons 2 and 3 of the gene). The two exons were amplified from the genomic DNA of 36 primary tumour samples and from eight lung carcinoma cell lines using primers sited within the adjacent introns. The PCR products were subjected to heteroduplex analysis using a Transgenomic WAVE system. With the exception of four samples that were heterozygous for a cSNP within exon 3 (Figure 2

), no traces suggestive of mutation were obtained from any of the tumours. We therefore conclude that mutational activation or inactivation of this gene is either rare or does not occur in NSCLC.

### Immunohistochemistry

Having previously demonstrated that the S100A2 transcript and protein are strongly expressed in tumour cells compared to normal lung, we sought to examine protein levels in the independent patient series. In addition to examining expression in normal and tumour tissue in this series, we also sought to examine expression in relation to preneoplasia, histology, and clinical outcome, in an attempt to further characterise the expression of S100A2 in NSCLC. Tumour and matching normal tissue was available from 48 NSCLC patients in the independent series. In addition, six bronchial sites distant from the primary tumour, were analysable for 20 of these patients. All tumours and non-tumour sites were investigated for S100A2 expression. Bronchial epithelia for each non-tumour site were graded (by our pathologist: MG) according to the degree of apparent histological abnormality into: normal, hyperplastic (basal cell and goblet cell), metaplastic, dysplastic or carcinoma *in situ* (CIS). Staining was assessed as described in Materials and Methods.

**Figure 3:**
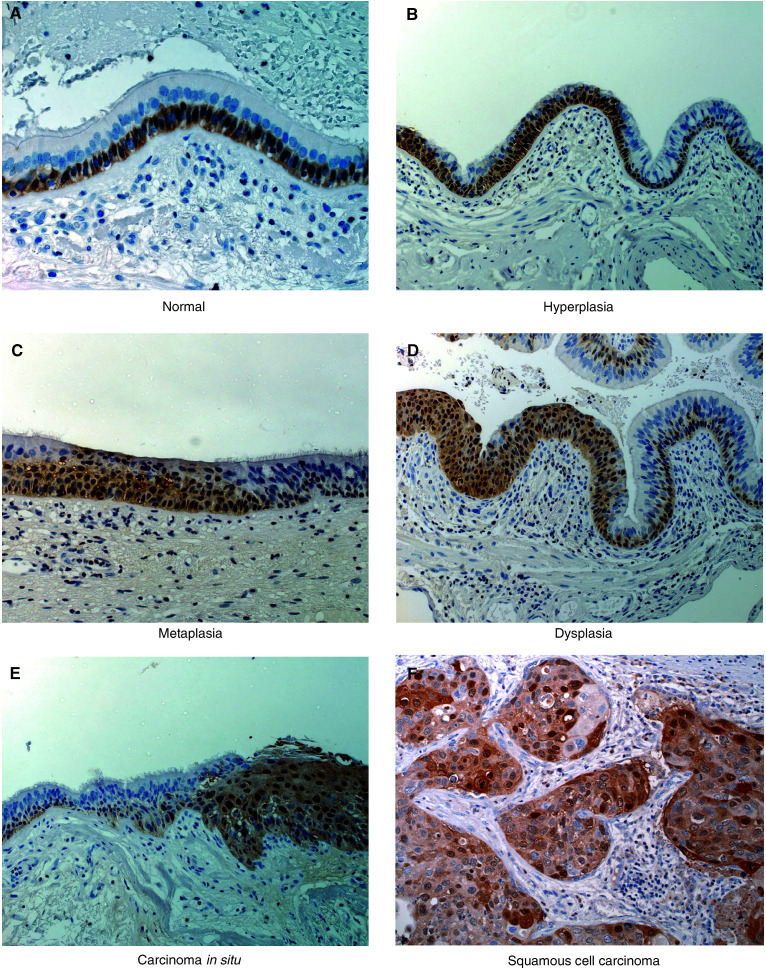
Immunohistochemical analysis of S100A2 expression in NSCLC and normal lung tissue. S100A2 is expressed strongly in the nucleus and also generally in the cytoplasm of basal epithelial cells of normal tissue but it is generally not detectable in histologically normal apical epithelial cells (**A**). Intense nuclear and cytoplasmic staining of the tumour cells is seen in positive tumours (**F**). Stromal cells are negative (**A**–**F**). However, a small number of hyperplastic epithelia show strong nuclear/cytoplasmic expression of S100A2 in the apical differentiated cells (**B**). The remaining panels show preneoplastic epithelial lesions, which commonly show strong staining for S100A2. The flanking histologically normal epithelia in the metaplasia, dysplasia and CIS panels show a more typical expression pattern.

Of the 48 tumour sections, 47 were analysable (one sample was rejected as highly necrotic). A total of 29 out of these 47 tumours (62%) strongly expressed S100A2 either in the nucleus (26 out of 47) or the cytoplasm (28 out of 47). Three tumours showed exclusively cytoplasmic staining with one showing exclusively nuclear staining: 25 out of 47 lesions therefore demonstrated staining in both compartments (see Figure 3

for representative images of staining). Squamous cell lesions were more likely to be positive than adenocarcinomas (nuclear *P*=0.0015, cytoplasmic *P*=0.0046: Table 2A

**Table 2:** Expression of S100A2 (*n*=114 sites: 101 non-neoplastic, normal and hyperplastic, and 13 preinvasive)


	**Nuclear**		**Cytoplasmic**	
**Tumours**	**Negative**	**Positive**	**Negative**	**Positive**
**(A) Expression pattern of S100A2 across a series of 47 primary NSCLCs^a^**				
Adenocarcinoma	15	9	14	10
Large cell	1	1		
LCNEC	2	2		
NSCLC undifferentiated	1	1		
Squamous cell	3	16	3	16
				

**Apical epithelial sites**				
**(B) Association of S100A2 expression of the apical part of the epithelium with histology (*n*=114 sites: 101 non-neoplastic, normal and hyperplastic, and 13 preinvasive)^b^**				
Normal	45	1	46	0
Hyperplastic (goblet/basal)	46	9	46	9
Metaplasia/dysplasia/CIS	5	8	7	6

a While there was a strong bias towards positive staining in squamous cell carcinomas, a significant fraction of adenocarcinomas also strongly expressed the gene.

b All histologically normal sites but one showed a consistent staining pattern of exclusively basal cell staining. Preneoplastic sites from individual patients showed strong expression. Apical cell staining was predominantly related to the histological state of the tissue and was not consistent for any patient across all non-neoplastic sites analysed.

), but we failed to detect a correlation between staining levels and overall survival or between staining and smoking history. This is perhaps not surprising given the small sample size. Although the RT–PCR and IHC assays measure slightly different qualities of gene expression and are performed on sections from different areas of the primary tumour, there was a good correlation between the tumours scored as positive and negative for strong S100A2 expression (*P*<0.0003).

Bronchial epithelial sites were identified in 117 separate sections from 20 patients. The majority of the differentiated epithelial cells in the normal lung did not express S100A2 (hence, the lower levels recorded by RT–PCR, Western and microarray analyses of normal lung tissue). However, strong nuclear staining of the basal epithelial cells was apparent in the overwhelming majority of both normal and other (hyperplastic/metaplastic/dysplastic/CIS) sites examined (97%, 113 out of 117, Figure 3) and therefore most likely represents a normal pattern of S100A2 expression for this cell type. Basal cell cytoplasmic staining was also commonly present (79%, 93 out of 117 sites). In contrast, the apical cells of histologically normal bronchial epithelia were generally negative for S100A2 expression, with only a single site (2%, one out of 46) showing strong (nuclear) staining (Table 2). As the degree of tissue disorder increased, so did the frequency with which lesions stained for S100A2 with: 16% (nine out of 55) of hyperplastic and 62% (eight out of 13) of preneoplastic (metaplasia, dysplasia, CIS) lesions showing strong nuclear staining (*P*<0.0001). Sites were classified as non-neoplastic (normal, hyperplastic) and preneoplastic. Apical cell nuclear and cytoplasmic staining was more commonly found in preneoplastic/preinvasive (eight out of 13, six out of 13) compared to non-neoplastic (10 out of 101, nine out of 101) epithelial sites (Table 2B; *P*<0.001).

### Allelic imbalance and allele-specific expression analysis

Unequal expression from the two alleles of an over-represented, nonimprinted autosomal gene in a tumour (allelic expression imbalance or AEI) may reflect either genomic damage events such as chromosomal translocation or point mutation or it may indicate allele-specific epigenetic alteration involving DNA methylation or histone deacetylation. The observation of tumour AEI is therefore consistent with the hypothesis that the overexpression of a particular gene is causally linked to neoplasia (Ratschiller *et al*, 2003).

**Figure 4:**
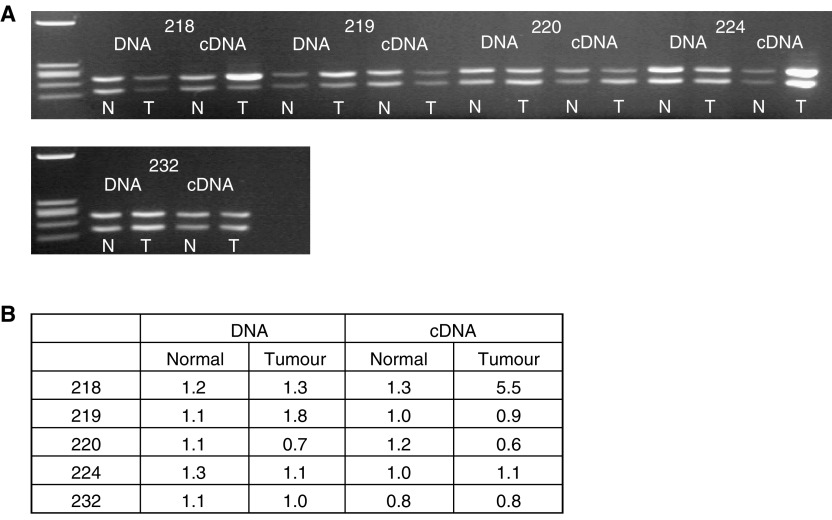
RFLP-cmRT–PCR and –PCR analysis of S100A2 in NSCLC. A region of the S100A2 gene containing a cSNP was amplified from DNA and cDNA by PCR and RT–PCR, respectively. Reaction products were digested with *Hinf*I and run on a 3% agarose gel (**A**). Products were also run on an Agilent bioanalyser DNA 1000 chip. Peak heights of the noncutting allele (upper product on the agarose gel) were divided by those of the cutting allele (lower band on agarose gel) to give a relative value for the cutting allele compared to the noncutting allele (**B**). Patient 218 shows an imbalance in the cDNA (allelic expression imbalance) of the tumour sample compared to the normal tissue.

A PCR-RFLP analysis was used to determine the relative contribution of the two S100A2 alleles to expression in our NSCLC patients. Results were quantified by Agilent Bioanalysis. Exon 3 encodes a rare cSNP (0.95/0.05) that can be used to assess the allelic balance in DNA and the corresponding allelic expression balance in cDNA. Typing of genomic DNA revealed that five out of the 46 patients investigated in the expression analysis were heterozygous for the cSNP. S100A2 was scored as over-represented in each of these lesions by cmRT–PCR. All patients showed balanced biallelic expression in the normal tissue samples. However, one patient (218) showed marked allelic expression imbalance in the tumour compared to the matched normal lung sample (Figure 4

). As allelic imbalance (AI) at the DNA level was not detected in this sample, this suggested that the strong expression seen was a consequence of upregulation of one chromosomal allele in the tumour. The other four patients all showed tumour allelic expression balances that approximated the allelic balance of the tumour DNA.

### ΔNp63 expression in NSCLC

S100A2 has recently been reported to be a target of the transcriptional activator ΔNp63 (Hibi *et al*, 2003), which is encoded by the TP73L gene. Bearing in mind this observation, the fact that four of five of the informative tumours showed strong biallelic S100A2 expression and considering that the products of TP73L were scored as significantly tumour over-represented in 24% (nine out of 37) of samples in our microarray analysis, we examined the expression of this gene in this independent NSCLC series. The initial microarray data suggested that S100A2 was also over-represented in eight out of nine of these particular tumours, but there were a further 10 lesions in which S1000A2 was strongly expressed in the apparent absence of TP73L transcript over-representation and one lesion in which TP73L was marginally increased with no apparently corresponding upregulation of S100A2. We therefore sought to validate these observations on a second set of tumours by a second technique.

**Figure 5:**
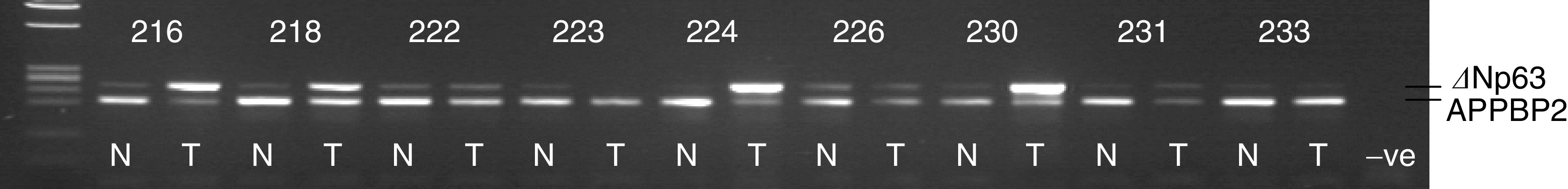
Expression of ΔNp63 in primary NSCLC. ΔNp63 transcripts were coamplified with transcripts of a control gene by comparative multiplex RT–PCR. Representative agarose gels are shown. Transcripts of the gene for APPBP2 were used as the control. N=normal bronchial epithelial tissue. T=NSCLC tissue. Numbers represent anonymised patient numbers.

RT–PCR analysis (not shown) was consistent with the ΔNp63 transcript being the predominantly spliced gene product in tumour tissues. Strong TP73L expression was not detected in the normal lung (Figure 5

). However, 23 out of 46 (50%) of patients showed over-representation of ΔNp63 in the tumour sample (see Figure 5 and Table 1). This expression correlated with S100A2 levels in 25 out of 40 patients (63%). However, six patients (15%) showed overexpression of S100A2 but not ΔNp63, and one out of 40 patients (2.5%) showed overexpression of ΔNp63 but not S100A2, indicating that other mechanisms in addition to ΔNp63 transcription are at least partly responsible for the high-level expression of S100A2 in tumour tissue.

## DISCUSSION

The detection and effective treatment of early stage disease is likely to dramatically improve survival rates for NSCLC patients. We have previously used cDNA microarray analysis to identify genes which are strongly expressed in tumour over normal tissue with the aim firstly of evaluating the involvement of such sequences in disease pathology and secondly, of investigating their possible utility as new diagnostic and/or therapeutic targets. Using this approach, we have demonstrated that S100A2 gene products are over-represented in the tumours of most NSCLC patients compared to matched normal lung tissue (Heighway *et al*, 2002). Surprisingly, based on a number of studies of gene expression, S100A2 is generally considered to be a tumour suppressor gene. In particular, using cell line models of the disease process, diminished expression has previously been reported to occur in lung cancer (Feng *et al*, 2001). Given the contradictory nature of our data, we therefore sought to further characterise S100A2 expression in an independent (from the microarray analysed) series of NSCLC patients, and to further investigate the role of S100A2 in early preneoplastic bronchial lesions.

Using semiquantitative RT–PCR and essentially confirming our earlier observations, 76% of NSCLC patients examined showed a two-fold or greater over-representation of S100A2 transcripts in their tumour sample compared to matched normal lung. S100A2 transcripts were consistently detected in normal tissues, but were present at much lower levels compared to tumour samples. Increased expression of the transcript appeared to be accompanied by increased protein expression and using IHC, 62% of tumours showed strong expression of S100A2 protein. The high levels of S100A2 were confirmed through Western analysis (not shown) of a subset of lesions with a second monoclonal antibody identical to that used by Feng *et al* (2001). Normal lung tissues were scored as negative for S100A2 expression (only one out of 46 showed positive staining) as most of the tissue stained negatively. However, strong nuclear and cytoplasmic staining was seen consistently in the basal cells of the bronchial epithelium. The other histologically normal cells of the lung including the columnar epithelia of the airways do not appear to express S100A2.

A similar pattern of expression for S100A2 has been reported in other tumour types. Specifically, the gene is expressed in the basal and parabasal cells of the epithelial layer of normal skin and in skin tumours (Shrestha *et al*, 1998; Park and Min 2003). Expression in normal squamous laryngeal epithelium and in laryngeal squamous cell carcinoma has also been reported (Lauriola *et al*, 2000). Furthermore, this expression was strictly associated with cytokeratins 14 and 17, which are markers of basal and parabasal cells in normal laryngeal epithelium (Van der Velden *et al*, 1997). These observations suggest that in normal epithelia, the expression of S100A2 is associated at least in some instances with multipotent populations of cells.

In this study, we have investigated S100A2 expression in histologically normal and hyperplastic epithelia and preneoplastic (metaplasia, dysplasia, CIS) lesions. We found that as the degree of preinvasive pathology increased, so did the frequency with which lesions stained for S100A2. Whereas, in normal epithelia, basal cells were positive and apical cells were negative for S100A2 expression, in some hyperplastic epithelia (16%) and the majority of preneoplastic (62%) epithelia, both basal cells and apical epithelial cells showed positive staining. The basal epithelia cell is a multipotent reserve cell which can differentiate into each of the mature cell types of the bronchial epithelium. Our analysis of the patterns of expression in normal tissue sections suggests that S100A2 expression should normally be downregulated in this differentiation process. Our observation of high levels of expression in preneoplastic regions suggests that this has not occurred, either coincidentally or pathologically in the cells of these lesions.

The identification of genetic damage affecting the S100A2 gene would add weight to the argument that this sequence is causally involved in neoplasia. Similarly, if this gene were a genuine tumour suppressor, we might expect to find coding sequence mutations inactivating function. We did not find any evidence of point mutation within the S100A2 coding sequence, and concluded that such an event is either rare or nonexistent in NSCLC. We have previously shown, using comparative multiplex PCR, that amplifications of the S100A2 gene in NSCLC are rare (Heighway *et al*, 2002), and we have confirmed this observation in the current patient series (data not shown). However, screening normal DNA samples, we were able to identify five patients who were heterozygous for a coding sequence SNP within exon 2 of the S100A2 gene. The matching tumour DNA for all five patients showed no evidence of allelic imbalance (AI) at this locus. We did however detect a marked allelic expression imbalance (AEI) in the tumour of one patient compared to their matched normal lung. This patient also showed strong expression of S100A2 in the tumour tissue. The expression imbalance is indicative of a *cis*-located DNA damage or modification event which results in the upregulation of one parental allele. This event could be a chromosomal translocation or a promoter mutation or an allelic methylation imbalance (AMI), any of which could lead to overexpression of the S100A2 transcript. Methylation differences within the S100A2 gene locus have previously been reported (Wicki *et al*, 1997) and it is possible that an aberrant lack of methylation of one parental allele could lead to inappropriate expression of the S100A2 gene in tumour tissue.

We have previously shown that p63 transcripts are over-represented in NSCLCs using cDNA microarray analysis (Heighway *et al*, 2002). In this study, we confirmed that the over-represented transcript is the ΔNp63 alternatively spliced gene product and that this is strongly expressed in 50% of tumours compared to normal lung. S100A2 has recently been reported to be a target of ΔNp63 (Hibi *et al*, 2003) a transcriptional activator. Overexpression of ΔNp63 in NSCLC has also been reported by Massion *et al* (2003), who also demonstrated that amplification of the p63 gene is an early event in lung cancer development. Interestingly, they also showed that p63 was strongly expressed in the basal cells of the normal bronchial epithelium. Therefore, one *trans*-acting mechanism whereby S100A2 may be overexpressed in NSCLC might be upregulation by ΔNp63. Although ΔNp63 expression correlated well with S100A2 expression – in that tumours strongly expressing TP73L tended to strongly express S100A2 – there was not an exact correlation. Specifically, while all but one of the tumours in both independent series (microarray, RT–PCR) that strongly expressed ΔNp63 coexpressed S100A2, there were a number of tumours (10 out of 37 and six out of 40, respectively) that showed over-representation of S100A2, in the apparent absence of ΔNp63 elevation. We therefore conclude that overexpression of ΔNp63 transcripts may explain S100A2 overexpression in some cases of NSCLC, but that this is probably not the only mechanism involved. While the allele-specific expression data presented support this idea, further cell-by-cell analysis of the level of S100A2 transcription in relation to the level of functional ΔNp63 protein would be needed to confirm this hypothesis.

Interestingly, although many adenocarcinomas (10 out of 24) did express S100A2, we found that expression correlated more closely with a squamous histology (16 out of 19). S100A2 is located within the epidermal differentiation complex (EDC), on chromosome 1q21 (South *et al*, 1999), and has been associated with squamous cell differentiation (Xia *et al*, 1997; Shrestha *et al*, 1998). The strong correlation with a squamous histology would perhaps suggest that S100A2 is functionally linked to the squamous phenotype. However, many lung adenocarcinomas and other nonsquamous cancers (for example, gastric carcinomas) strongly express S100A2. Squamous cell lung carcinomas tend to be centrally located, arising in a major airway, from a component of the bronchial epithelium. Lung adenocarcinomas can form centrally but they tend to be associated with the epithelia of the peripheral airways. Recently, it has been suggested that adenocarcinoma can be classified into two distinct subtypes on the basis of gene expression patterns (Bhattacharjee *et al*, 2001; Garber *et al*, 2001). These two subtypes may be delineated by TTF (thyroid transcription factor)-1 expression, which is a lineage marker for peripheral airway cells, including type I and II pneumocytes (Yatabe *et al*, 2004). Interestingly, TTF-1 expression in adenocarcinoma is inversely correlated with expression of maspin (Kobayashi *et al*, 2004), which we have previously shown to be tightly restricted to basal cells of the bronchial epithelium in normal lung (Smith *et al*, 2003). Normal alveoli do not express S100A2. Taken together, these observations suggest that squamous cell carcinomas that are generally positive for S100A2 expression and S100A2 positive adenocarcinomas may arise either from the multipotent basal cells of the bronchial epithelium or else from a stem cell progenitor which expresses this and other tumour-associated genes (maspin, ΔNp63). S100A2/maspin-negative adenocarcinomas may similarly arise from such multipotent progenitors, in which case they would represent a clonal lesion more closely associated with the expression pattern of a differentiated peripheral airway cell or else they may actually arise from such a cell. Such a model would suggest that tumour S100A2 expression is related to the expression pattern of the cell-type of origin and not simply a characteristic of squamous cell differentiation.

The function of S100A2 remains unknown. It is widely thought to be a tumour suppressor (Lee *et al*, 1992; Wicki *et al*, 1997; Nagy *et al*, 2001). However, S100A2 is strongly expressed in many tumours (Villaret *et al*, 2000; Hough *et al*, 2001; El-Rifai *et al*, 2002). It is also expressed in regenerative hyperplasia of keratinocytes and is active psoriasis (Xia *et al*, 1997), which are characterised by increased signal transduction through the cErbB family of receptor tyrosine kinases (Stoll *et al*, 1997). Activation of the EGF receptor, which is mutated in primary lung cancers (Lynch *et al*, 2004) selectively stimulates S100A2 gene expression, at the transcriptional level (Stoll *et al*, 1998). EGFR-dependent S100A2 expression in keratinocytes is thought to correlate with proliferation (Xia *et al*, 1997) but only in cells committed to squamous differentiation. This is consistent with the observation that expression of S100A2 in psoriatic skin occurs in the supra-basal epithelial layers. However, the protein is expressed consistently at high levels in the normal basal epithelial cells of the airways, which are generally in a nonproliferating state. S100A2 expression in this multipotent cell type is therefore unlikely to be linked to a cause or effect of commitment to a squamous cell lineage and must serve some other function, perhaps one associated with some aspect of self-renewal.

A number of genes have recently been shown to be expressed strongly in tumours, preneoplastic bronchial lesions and in the normal multipotent basal epithelial cells of the airway: examples include S100A2, TP73L and SERPINB5. Similarly, basal cell markers such as cytokeratin 17 and 19 are among the most frequently expressed genes in NSCLC tissues (Heighway *et al*, 2002: for lesion-by-lesion detail see http://www.roycastle.org/resea rch/presentations.htm). Such observations perhaps lend weight to the hypothesis that tumours arise directly from a multipotent or stem cell component of the epithelium. One possibility is that a significant fraction of tumour over-represented genes are normally inactivated as such multipotent stem cell (like) cells differentiate, perhaps (as has been implied for SERPINB5) by promoter methylation. The unusual expression of genes like SERPINB5 and S100A2 in differentiated preinvasive cells (and subsequently in tumours) may therefore indicate that a failure to appropriately inactivate stem cell-associated sequences, perhaps by epigenetic modification, is an important proneoplastic mechanism. The wider exploration of tumour expression data in this context might therefore refine our understanding of the origins of neoplasia.

Taken together, the observations presented are consistent with a direct role for S100A2 in bronchial neoplasia. Our failure to identify any inactivating mutations in this gene fails to support the argument that the sequence is a tumour suppressor in the lung. Further functional analysis will be required to determine whether S100A2 might represent a novel therapeutic or diagnostic target in early lung cancer.

## References

[bib1] Bhattacharjee A, Richards WG, Staunton J, Li C, Monti S, Vasa P, Ladd C, Beheshti J, Bueno R, Gillette M, Loda M, Weber G, Mark EJ, Lander ES, Wong W, Johnson BE, Golub TR, Sugarbaker DJ, Meyerson M (2001) Classification of human lung carcinomas by mRNA expression profiling reveals distinct adenocarcinoma subclasses. Proc Natl Acad Sci USA 98: 13790–13795 11707567 10.1073/pnas.191502998 PMC61120

[bib2] Bray F, Sankila R, Ferlay J, Parkin DM (2002) Estimates of cancer incidence and mortality in Europe in 1995. Eur J Cancer 38: 99–166 11750846 10.1016/s0959-8049(01)00350-1

[bib3] Donato R (1999) Functional roles of S100 proteins, calcium-binding proteins of the EF-hand type. Biochim Biophys Acta 1450: 191–231 10395934 10.1016/s0167-4889(99)00058-0

[bib4] El-Rifai W, Moskaluk CA, Abdrabbo MK, Harper J, Yoshida C, Riggins RJ, Frierson Jr HF, Powell SM (2002) Gastric cancers overexpress S100A calcium-binding proteins. Cancer Res 62: 6823–6826 12460893

[bib5] Feng G, Xu X, Youssef EM, Lotan R (2001) Diminished expression of S100A2, a putative tumor suppressor, at earlystage of human lung carcinogenesis. Cancer Res 61: 7999–8004 11691825

[bib6] Garber ME, Troyanskaya OG, Schluens K, Petersen S, Thaesler Z, Pacyna-Gengelbach M, van de Rijn M, Rosen GD, Perou CM, Whyte RI, Altman RB, Brown PO, Botstein D, Petersen I (2001) Diversity of gene expression in adenocarcinoma of the lung. Proc Natl Acad Sci USA 98: 13784–13789 11707590 10.1073/pnas.241500798 PMC61119

[bib7] Heighway J, Betticher DC, Knapp T, Hoban PR (2003) Comparative multiplex PCR and allele specific expression analysis in human lung cancer: tools to facilitate target identification. Lung Cancer, Ed: B Driscoll. Methods Mol Med 75: 291–304 12407748 10.1385/1-59259-324-0:291

[bib8] Heighway J, Knapp T, Boyce L, Brennand S, Field JK, Betticher DC, Ratschiller D, Gugger M, Donovan M, Lasek A, Rickert P (2002) Expression profiling of primary non-small cell lung cancer for target identification. Oncogene 21: 7749–7763 12400018 10.1038/sj.onc.1205979

[bib9] Hibi K, Fujitake S, Takase T, Kodera Y, Akiyama S, Shirane M, Nakao A (2003) Identification of S100A2 as a target of the DeltaNp63 oncogenic pathway. Clin Cancer Res 9: 4282–4285 14519656

[bib10] Hough CD, Cho KR, Zonderman AB, Schwartz DR, Morin PJ (2001) Coordinately up-regulated genes in ovarian cancer. Cancer Res 61: 3869–3876 11358798

[bib11] Ilg EC, Schäfer BW, Heizmann CW (1996) Expression pattern of S100 calcium-binding proteins in human tumors. Int J Cancer 68: 325–332 8903474 10.1002/(SICI)1097-0215(19961104)68:3<325::AID-IJC10>3.0.CO;2-7

[bib12] Kobayashi K, Nishioka M, Kohno T, Nakamoto M, Maeshima A, Aoyagi K, Sasaki H, Takenoshita S, Sugimura H, Yokota J (2004) Identification of genes whose expression is upregulated in lung adenocarcinoma cells in comparison with type II alveolar cells and bronchiolar epithelial cells *in vivo*. Oncogene 23: 3089–3096 14755238 10.1038/sj.onc.1207433

[bib13] Lauriola L, Michetti F, Maggiano N, Galli J, Cadoni G, Schafer BW, Heizmann CW, Ranelletti FO (2000) Prognostic significance of the Ca(2+) binding protein S100A2 in laryngeal squamous-cell carcinoma. Int J Cancer 89: 345–349 10956408 10.1002/1097-0215(20000720)89:4<345::aid-ijc5>3.0.co;2-t

[bib36] Lee SW, Tomasetto C, Sager R (1991) Positive selection of candidate tumor-suppressor genes by subtractive hybridization. Proc Natl Acad Sci USA 88: 2825–2829 1849277 10.1073/pnas.88.7.2825 PMC51332

[bib14] Lee SW, Tomasetto C, Swisshelm K, Keyomarsi K, Sager R (1992) Down-regulation of a member of the S100 gene family in mammary carcinoma cells and re-expression by azadeoxycytidine treatment. Proc Natl Acad Sci USA 89: 2504–2508 1372446 10.1073/pnas.89.6.2504 PMC48687

[bib15] Liu D, Rudland PS, Sibson DR, Platt-Higgins A, Barraclough R (2000) Expression of calcium-binding protein S100A2 in breast lesions. Br J Cancer 83: 1473–1479 11076656 10.1054/bjoc.2000.1488 PMC2363420

[bib16] Lynch TJ, Bell DW, Sordella R, Gurubhagavatula S, Okimoto RA, Brannigan BW, Harris PL, Haserlat SM, Supko JG, Haluska FG, Louis DN, Christiani DC, Settleman J, Haber DA (2004) Activating mutations in the epidermal growth factor receptor underlying responsiveness of non-small-cell lung cancer to gefitinib. N Engl J Med 50: 2129–2139 10.1056/NEJMoa040938 15118073

[bib17] Maelandsmo GM, Florenes VA, Mellingsaeter T, Hovig E, Kerbel RS, Fodstad O (1997) Differential expression patterns of S100A2, S100A4 and S100A6 during progression of human malignant melanoma. Int J Cancer 74: 464–469 9291441 10.1002/(sici)1097-0215(19970822)74:4<464::aid-ijc19>3.0.co;2-9

[bib18] Massion PP, Taflan PM, Jamshedur Rahman SM, Yildiz P, Shyr Y, Edgerton ME, Westfall MD, Roberts JR, Pietenpol JA, Carbone DP, Gonzalez AL (2003) Significance of p63 amplification and overexpression in lung cancer development and prognosis. Cancer Res 63: 7113–7121 14612504

[bib19] Nagy N, Brenner C, Markadieu N, Chaboteaux C, Camby I, Schafer BW, Pochet R, Heizmann CW, Salmon I, Kiss R, Decaestecker C (2001) S100A2, a putative tumor suppressor gene, regulates *in vitro* squamous cell carcinoma migration. Lab Invest 81: 599–612 11304580 10.1038/labinvest.3780269

[bib20] O'Donovan MC, Oefner PJ, Roberts SC, Austin J, Hoogendoorn B, Guy C, Speight G, Upadhyaya M, Sommer SS, McGuffin P (1998) Blind analysis of denaturing high-performance liquid chromatography as a tool for mutation detection. Genomics 52: 44–49 9740670 10.1006/geno.1998.5411

[bib21] Park HR, Min SK (2003) Expression of S100A2 and S100B proteins in epithelial tumors of the skin. J Cutan Pathol 30: 373–378 12834486 10.1034/j.1600-0560.2003.00081.x

[bib22] Pedrocchi M, Schafer BW, Mueller H, Eppenberger U, Heizmann CW (1994) Expression of Ca(2+)-binding proteins of the S100 family in malignant human breast-cancer cell lines and biopsy samples. Int J Cancer 57: 684–690 8194876 10.1002/ijc.2910570513

[bib23] Ratschiller D, Heighway J, Gugger M, Kappeler A, Pirnia F, Schmid R, Borner MM, Betticher DC (2003) Cyclin D1 overexpression in bronchial epithelia of patients with lung cancer is associated with smoking and predicts survival. J Clin Oncol 21: 2085–2093 12775733 10.1200/JCO.2003.03.103

[bib24] Shrestha P, Muramatsu Y, Kudeken W, Mori M, Takai Y, Ilg EC, Schäfer B W, Heinzmann CW (1998) Localisation of Ca^2+^-binding S100 proteins in epithelial tumours of the skin. Vichows Arch 432: 53–59 10.1007/s004280050134 9463588

[bib25] Smith SL, Watson SG, Ratschiller D, Gugger M, Betticher DC, Heighway J (2003) Maspin – the most commonly-expressed gene of the 18q21.3 serpin cluster in lung cancer – is strongly expressed in pre-neoplastic bronchial lesions. Oncogene 22: 8677–8687 14647462 10.1038/sj.onc.1207127

[bib26] South AP, Cabral A, Ives JH, James CH, Mirza G, Marenholz I, Mischke D, Backendorf C, Ragoussis J, Nizetic D (1999) Human epidermal differentiation complex in a single 2.5 Mbp long continuum of overlapping DNA cloned in bacteria integrating physical and transcript maps. J Invest Dermatol 112: 910–918 10383738 10.1046/j.1523-1747.1999.00613.x

[bib27] Stoll SW, Kansra S, Elder JT (1997) Keratinocyte outgrowth from human skin explant cultures is dependent upon p38 signaling. Wound Repair Regen 11: 346–353 10.1046/j.1524-475x.2003.11506.x 12950638

[bib28] Stoll SW, Zhao X, Elder JT (1998) EGF stimulates transcription of CaN19 (S100A2) in HaCaT keratinocytes. J Invest Dermatol 111: 1092–1097 9856822 10.1046/j.1523-1747.1998.00402.x

[bib29] Travis WD, Colby TV, Corrin B, Shimosato Y, Brambilla E (1999) Histological Typing of Lung and Pleural Tumours (World Health Organisation: International Classification of tumours). Springer-Verlag: Berlin, GmbH & Co. K: Heidelberg, ISBN: 3540652191

[bib30] Van der Velden LA, Schaafsma HE, Manni JJ, Rutter DJ, Raemakers FCS, Kuiypers W (1997) Cytokeratin and vimentin expression in normal epithelium and squamous cell carcinomas of the larynx. Europ Arch Otolaryngol 254: 376–383 10.1007/BF01642554 9332893

[bib31] Villaret DB, Wang T, Dillon D, Xu J, Sivam D, Cheever MA, Reed SG (2000) Identification of genes overexpressed in head and neck squamous cell carcinoma using a combination of complementary DNA subtraction and microarray analysis. Laryngoscope 110: 374–381 10718422 10.1097/00005537-200003000-00008

[bib32] Whang-Peng J, Knutsen T, Gazdar A, Steinberg SM, Oie H, Linnoila I, Mulshine J, Nau M, Minna JD (1991) Nonrandom structural and numerical chromosome changes in non-small-cell lung cancer. Genes Chromosomes Cancer 3: 168–188 1651103 10.1002/gcc.2870030303

[bib33] Wicki R, Franz C, Scholl FA, Heizmann CW, Schafer BW (1997) Repression of the candidate tumor suppressor gene S100A2 in breast cancer is mediated by site-specific hypermethylation. Cell Calcium 22: 243–254 9481475 10.1016/s0143-4160(97)90063-4

[bib34] Xia L, Stoll SW, Lieber M, Ethier SP, Carey T, Esclamado R, Carroll W, Johnson TM, Elder JT (1997) CaN19 expression in benign and malignant hyperplasias of the skin and oral mucosa: evidence for a role in regenerative differentiation. Cancer Res 57: 3055–3062 9230222

[bib35] Yatabe Y, Mitsudomi T, Takahashi T (2004) Maspin expression in normal lung and non-small-cell lung cancers: cellular property-associated expression under the control of promoter DNA methylation. Oncogene 23: 4041–4049 15048080 10.1038/sj.onc.1207557

